# No Evidence for Semantic Prediction Deficits in Individuals With Cerebellar Degeneration

**DOI:** 10.1162/nol_a_00083

**Published:** 2024-08-15

**Authors:** Maedbh King, Sienna Bruinsma, Richard B. Ivry

**Affiliations:** Department of Psychology, University of California, Berkeley, CA, USA; Helen Wills Neuroscience Institute, University of California, Berkeley, CA, USA

**Keywords:** cerebellar degeneration, cerebellum, internal model, prediction, semantic prediction

## Abstract

Cerebellar involvement in language processing has received considerable attention in the neuroimaging and neuropsychology literatures. Building off the motor control literature, one account of this involvement centers on the idea of internal models. In the context of language, this hypothesis suggests that the cerebellum is essential for building semantic models that, in concert with the cerebral cortex, help anticipate or predict linguistic input. To date, supportive evidence has primarily come from neuroimaging studies showing that cerebellar activation increases in contexts in which semantic predictions are generated and violated. Taking a neuropsychological approach, we put the internal model hypothesis to the test, asking if individuals with cerebellar degeneration (*n* = 14) show reduced sensitivity to semantic prediction. Using a sentence verification task, we compare reaction time to sentences that vary in terms of cloze probability. We also evaluated a more constrained variant of the prediction hypothesis, asking if the cerebellum facilitates the generation of semantic predictions when the content of a sentence refers to a dynamic rather than static mental transformation. The results failed to support either hypothesis: Compared to matched control participants (*n* = 17), individuals with cerebellar degeneration showed a similar reduction in reaction time for sentences with high cloze probability and no selective impairment in predictions involving dynamic transformations. These results challenge current theorizing about the role of the cerebellum in language processing, pointing to a misalignment between neuroimaging and neuropsychology research on this topic.

## INTRODUCTION

Anatomical, neuropsychological, and neuroimaging work over the last 35 years has implicated the cerebellum in functions extending well beyond the motor domain. Throwing down the gauntlet for a “cognitive cerebellar revolution,” Leiner et al. (1986) highlighted the expansion of the cerebellum across vertebrate evolution and, in particular, a parallel expansion of the neocerebellum and prefrontal cortex. They hypothesized that this pattern likely reflected extensive communication between the cerebellum and cortical association regions, connections that could support mental coordination in a manner analogous to how cerebellocortical connectivity supported motor coordination. Over the following decades, the neuropsychological, neuroimaging, and brain stimulation literatures have provided ample evidence in line with this general perspective. Behavioral impairments on a variety of cognitive and affective tasks have been described in individuals with cerebellar dysfunction (Ivry & Fiez, 2000; Kansal et al., 2017; Schmahmann & Sherman, 1998; Sokolov et al., 2017), and activation is consistently observed in the cerebellum during a broad range of tasks that is not readily related to motor preparation or production (King et al., 2019; Stoodley & Schmahmann, 2009b).

One prominent domain of interest is language. In their seminal PET (positron emission tomography) study, Petersen et al. (1989) used a subtractive logic to identify brain regions involved with different linguistic processes. Their results revealed prominent activation of the right cerebellum in a contrast designed to show regions involved in semantic retrieval while controlling for articulatory preparation. This observation has been confirmed in many subsequent neuroimaging studies investigating semantic retrieval and prediction, with the activation centered in right medial-to-lateral Crus I/II (Fiez et al., 1996; Lesage et al., 2017; Moberget et al., 2016). The neuropsychology literature presents a more problematic picture. While speech dysarthria is a prominent feature in some forms of cerebellar ataxia (Ackermann & Hertrich, 1994; Richter et al., 2007), the evidence is mixed in terms of whether cerebellar dysfunction, at least when emerging in adulthood, disrupts semantic processing. In a widely cited case study by Fiez et al. (1992), a patient with a right inferior cerebellar lesion had marked difficulty on a semantic generation task, either coming up with an inappropriate semantic associate to a target noun or failing to follow instructions and simply repeating the target word. However, subsequent studies failed to find group-level differences on similar tasks (Gasparini et al., 1999; Helmuth et al., 1997; Richter et al., 2004; Riva, 1998). While it is possible that these null results reflect the heterogeneity in the patient samples, it may be that semantic generation tasks are not sensitive to cerebellar-related language impairments or optimal for assessing computations performed by the cerebellum in semantic processing.

Theoretically, researchers have turned to the sensorimotor control literature in considering how the cerebellum may contribute to semantic processing. One key idea has centered on the notion of prediction and, specifically, how the cerebellum may generate internal models to anticipate future input based on the current context (Ito, 2008; Wolpert et al., 1998). In the motor context, an internal model may represent the dynamic properties of an object (e.g., body part, tool), allowing the agent to anticipate the sensory consequences of an action within a particular environment (Wolpert et al., 1995). Extending this idea to language, an internal model would operate on abstract semantic knowledge and input from the current discourse to generate semantic expectancies; for example, to anticipate the speaker’s next utterance or anticipate when the speaker would pause to facilitate fluid turn taking (Lesage et al., 2012, 2017; Moberget et al., 2014).

Semantic verification tasks have provided one test bed of the internal model hypothesis. In an functional magnetic resonance imaging (fMRI) study, Moberget et al. (2014) compared the hemodynamic response to three types of sentences: (1) congruent, in which the last word was highly predictable given the preceding, base phase; (2) incongruent, in which the last word violated a prediction established by the base phase; and (3) scrambled, in which the words in the base phrase were randomly shuffled, thus precluding the generation of a prediction. An analysis time-locked to the appearance of the last word revealed two key cerebellar results. First, violations of predictions produced bilateral activation encompassing posterolateral Crus I/II compared to either congruent or scrambled sentences. Second, compared to scrambled sentences, congruent sentences produced activation in a more circumscribed portion of posterolateral cerebellum, now restricted to the right cerebellar hemisphere only. Thus, these data are in accord with the hypothesis that the cerebellum not only processes violations of predictions (which might suggest a role in error-detection/error-correction), but also is engaged in the generation of predictions. Lesage et al. (2017) reported similar effects in an event-related fMRI design that manipulated sentence predictability. Not only did they find that activity in the right posterolateral cerebellum correlated with the predictability of the target word, but they also demonstrated that this same region was engaged in phonological, but not semantic or orthographic processing.

Behavioral assays of cerebellar involvement in language have come from two sources, noninvasive brain stimulation targeted at the cerebellum in healthy individuals (Argyropoulos, 2016; D’Mello et al., 2017; Gatti et al., 2021; Lesage et al., 2012) or experiments involving individuals with cerebellar pathology (Alexander et al., 2012; Kansal et al., 2017; Stoodley & Schmahmann, 2009a). Lesage et al. (2012) instructed participants to shift their gaze to a peripheral object that was predicted by the semantic content of a spoken sentence. After repetitive transcranial magnetic stimulation (rTMS) was applied targeting right posterolateral cerebellum, participants took longer to fixate on the predicted object. In contrast, rTMS had no effect when the semantic content did not predict the target object, and the selective effect on predictive looking was not observed when the rTMS was directed at the vertex, a control stimulation location.

Along the same lines, D’Mello et al. (2017) showed that, after transcranial direct current stimulation (tDCS) over the right lateral cerebellum, the blood oxygen level dependent (BOLD) response in the cerebellum was selectively elevated in response to predictive sentences. Interestingly, the change in activation was not accompanied by a change in behavior. Participants were faster to verify predictable sentences compared to non-predictive, and this advantage was similar in the tDCS and control conditions. Similarly, Moberget et al. (2016) failed to observe any difference in behavior between individuals with cerebellar pathology and matched control participants. Thus, there is a mismatch between the imaging/neurostimulation and neuropsychology results. Consistent with the internal model hypothesis, increased activation is observed in the right posterolateral cerebellum when the semantic content of a sentence supports the formation of a prediction. However, the evidence is mixed in terms of whether lesions encompassing this region disrupts the ability to make semantic-based predictions. More generally, the neuropsychological literature fails to provide strong evidence of a role of the cerebellum in semantic processing. While individuals with cerebellar degeneration (CD) consistently show impairment in tests of fluency or word completion (Alexander et al., 2012; Kansal et al., 2017; Stoodley & Schmahmann, 2009a, 2009b), these tasks heavily tax processes associated with cognitive control.

In the present study, we set out to conduct a new neuropsychological test of the internal model hypothesis, comparing the performance of individuals with cerebellar degeneration and matched controls on a sentence verification task. Similar to our earlier studies (Moberget et al., 2014, 2016), participants made a two-alternative forced choice response on a semantic prediction task, indicating if each sentence was meaningful or meaningless. We emphasized reaction time in the current study, under the assumption that this would provide a more sensitive measure of performance than accuracy. We manipulated the strength of the prediction of the final word in the sentence given its base phrase. To this end, we manipulated cloze probability, a common linguistic metric that quantifies the context-dependent predictability of a word. Our main analysis focused on the meaningful sentences. We expected that control participants would have faster reaction times in response to target words with high cloze probability compared to target words with low cloze probability (Staub et al., 2015), reflecting the benefits of predictive processing when the base phrase allows the participant to anticipate the target word. If the integrity of the cerebellum is important for generating semantic predictions, we expect that the CD group would show an attenuated effect of cloze probability: Specifically, the CD group should show a reduced benefit to target words with high cloze probability.

As a secondary test of the prediction hypothesis, we analyzed reaction times for the meaningless sentences to examine how the two groups responded to violations of semantic predictions. This analysis is problematic for two reasons. First, these sentences entail two types of prediction violations. There is the general semantic violation given that the final word results in a nonsensical sentence. And, at least for the high cloze sentences, there is a prediction violation in that the final word does not match the expected word. Second, we did not have a strong a priori hypothesis here, even for the controls. Reaction times may be faster when the base phrase suggests a strong prediction since the violation would be easier to detect. Alternatively, reaction times might be slower when the base phrase suggests a strong prediction since anticipation of the expected final word might cause interference in processing the target word. Nonetheless, the meaningless sentences provide a test bed to see if the CD and control groups showed different response time patterns to semantic prediction violations.

We also designed the experiment to test a second hypothesis concerning predictive functions of the cerebellum. Even in the motor domain, prediction is not the exclusive domain of the cerebellum; indeed, one could argue that most neural activity is fundamentally about prediction (Friston, 2009). This leads to the question of what might be constraints on cerebellar-dependent predictions. Various candidates have been proposed over the years, some that specify the task (e.g., sensory consequences of movements generated by the skeletal musculature; Day et al., 1998) or computational (e.g., timing, error-based learning; Keele & Ivry, 1990; Wolpert et al., 1998) domain. McDougle et al. (2022) have recently proposed that the cerebellum is essential for predictions that require a continuous representational transformation (CoRT). The key idea is that the cerebellum facilitates mental processing when an internal representation undergoes a continuous dynamic transformation but is not essential when the mental operations entail more discrete or transformations or maintenance of static representations. In our initial work on this problem, the CoRT constraint provided a parsimonious account of the pattern of impairments (and spared performance) in the domains of visual cognition and arithmetic. For example, individuals with CD were impaired in the rate at which they mentally manipulated a visual image (Shepard & Metzler, 1971), whereas they showed a normal processing rate in the iterative evaluation of a series of discrete spatial representations.

In the context of language, we hypothesize that a CoRT-like operation would be relevant when the semantic content of a sentence conveys a dynamic representational transformation. For example, the sentence “The man cut the rope with a pair of scissors” evokes a strong, dynamic mental simulation, with a conceptualization of the context (an agent, desiring to cut a rope), helping anticipate the final word. In contrast, the sentence “Spring was her favorite season of the year” is relatively static. One could say it requires an internal model, one that captures the idea that a person has their idiosyncratic preferences for a season. But this model does not evoke a dynamic simulation. Based on this reasoning, we also designed the experiment such that we could compare sentences that described dynamic transformations (CoRT-dependent) with those that described static situations (non-CoRT). Although we do not have an a priori prediction for the control participants on this dimension, we predicted that the CD group would be relatively impaired on the sentence verification task for the dynamic sentences.

## MATERIALS AND METHODS

**Figure 1.**
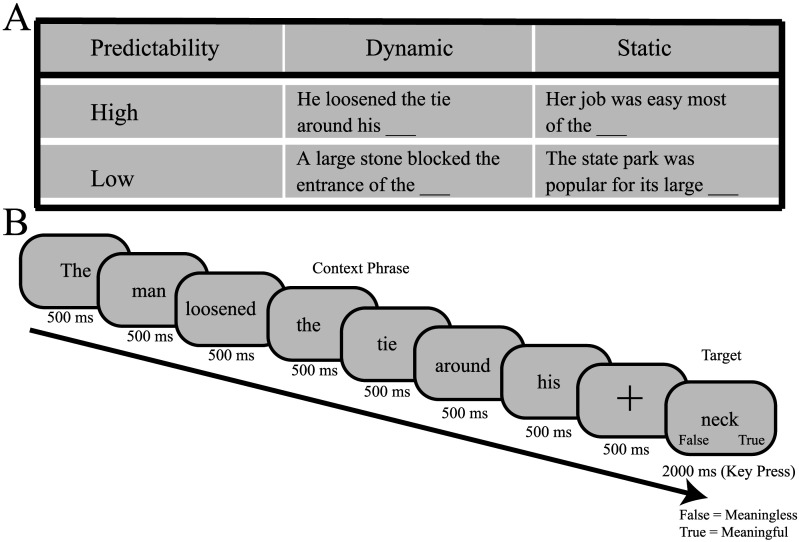
Task design. (A) Representative sentences for evaluating the internal and CoRT models. Sentence predictability is manipulated via high and low cloze probabilities, and representational transformations are manipulated via dynamic and static simulations. (B) Example trial. Each word of the stem (“The man loosened the tie around his”) is presented for 500 ms, followed by a fixation cross (500 ms). Participants then have 2,000 ms to make a key press indicating “true” or “false” on the target word (“neck”). True indicates a meaningful sentence, while false indicates a meaningless sentence.

We tested individuals with CD and matched controls on a semantic verification task. Participants were asked to determine whether a sentence was meaningful or meaningless based on the final word of the sentence. We manipulated two variables, prediction strength and dynamic context in a 2 × 2 design (Figure 1). All testing, including the neurological evaluation of the CD participants was conducted online.

We first describe the procedures involved in creating the stimulus materials for the study and then turn to the procedure for the main experiment. The study was approved by the institutional review board at the University of California, Berkeley.

### Construction and Evaluation of Stimuli

Sentences were drawn from two sentence databases, both of which included cloze probability ratings (Block & Baldwin, 2010; Peelle et al., 2020). Together, there are 3,583 sentences in the two databases. The 498 sentences in the Block and Baldwin database have a minimum cloze probability of 0.3. A cloze probability is calculated as the proportion of participants who give the same response to an incomplete sentence or passage (Kutas & Hillyard, 1984). We applied this same minimum criterion to the Peelle database, which excluded 923 of the 3,085 sentences in that database, yielding a total of 2,660 sentences in the initial inclusion stimulus set.

We enlisted raters to evaluate the degree to which these 2,660 sentences conveyed a dynamic event (CoRT-ness rating). We initially recruited undergraduate participants (*n* = 8, 4 female/4 male) through a research participant pool at the University of California, Berkeley. These individuals completed the rating procedure in the lab. However, with the onset of institutional restrictions related to the COVID-19 pandemic, we transitioned to online methods. For these ratings, we recruited participants (*n* = 61) through Prolific (https://www.prolific.co/), an online participant recruitment platform. We verified via self-report that the first language of all participants was English. Participants received monetary compensation for their time.

Participants were asked to rate each sentence in terms of how strongly the content evoked a dynamic event, using a 5-point Likert scale where 1 indicated *strongly disagree* (i.e., strongly non-CoRT) and 5 indicated *strongly agree* (i.e., strongly CoRT). Participants were provided examples of sentences conveying a strong dynamic event (e.g., “The man loosened the tie around his neck.”) and a weak dynamic event (e.g., “Her job was easy most of the time.”). As a primer, participants were given a list of five sentences with the correct ratings to ensure that they understood the goal of the task.

Each trial began with the presentation of a fixation cross. After 500 ms, a target sentence was displayed at the center of the screen, along with the instruction prompt at the bottom of the screen, “Does this sentence evoke strong, changing imagery?” Participants had 10 s to indicate their response using the number keys “1” to “5.” The fixation cross appeared as soon as the rating was registered. The in-person participants rated all 500 of the sentences in the Block & Baldwin data set. The stimulus order was randomized across individuals and breaks were provided every 180 trials. For the online participants, we used the Gorilla platform (https://gorilla.sc/) to run the experiment. The 2,160 sentences retained from the Peelle database were randomly divided into 12 sets of 180 sentences each. Each participant rated one set, with a break provided after 90 trials. The stimuli within a set were randomized across individuals.

For the final stimulus set, we used the cloze probability information and CoRT ratings to select 125 sentences for each of the four cells created by the factorial crossing of prediction and dynamics (Figure 1A). On the prediction dimension, sentences had to have a cloze probability between 0.3 and 0.5 to be selected as weak exemplars, or between 0.8 and 1.0 to be selected as strong exemplars. In this way, we created a similar cloze range for each level. For the dynamics dimension, sentences had to have a mean rating of less than 2 (static) or mean rating greater than 4 (dynamic). For high cloze sentences, dynamic conditions had an average CoRT rating of 4.214 (*SD* = 0.24), and static conditions had a rating of 1.616 (*SD* = 0.306). The average values of high cloze probabilities were roughly equivalent across dynamic (*M* = 0.885, *SD* = 0.053) and static (*M* = 0.878, *SD* = 0.049) ratings. For low cloze sentences, dynamic conditions had an average CoRT rating of 4.357 (*SD* = 0.298), and static conditions had an average rating of 1.655 (*SD* = 0.305). The average values of low cloze probabilities were roughly equivalent across dynamic (*M* = 0.407, *SD* = 0.058) and static (*M* = 0.398, *SD* = 0.057) ratings.

As a final step, we conducted a pilot of the semantic verification task, with participants making speeded responses to indicate if the sentence was meaningful or non-meaningful (detailed below). Participants (*n* = 77) were recruited via Prolific and tested across six blocks with the full set of 500 sentences, with a break provided approximately every 80 trials. Based on the results of the pilot study, we designed the main experiment to include five blocks each comprising 64 trials. We opted to reduce the number of blocks from six (in the pilot) to five (main experiment) to reduce the experiment time for our patient group. We divided the 500 pilot sentences into four conditions: (1) dynamic, high cloze; (2) dynamic, low cloze; (3) static, high cloze; (4) static, low cloze. Within each of those groups, we chose 80 sentences that had the lowest standard deviation across the pilot participants, resulting in a total of 320 sentences for the main experiment.

### Main Experiment

#### Participants

Seventeen individuals with CD and 19 age- and education-matched control participants were recruited, drawing on a patient database maintained by our lab. The database includes individuals recruited from ataxia support groups around the country and through advertisements posted on the National Ataxia Foundation webpage. Researchers from our group sent recruitment letters to support group leaders and these leaders shared our letters with their members. Interested individuals contacted the lab and after completing a series of screening procedures, were entered into the CD database if they met inclusion criteria (Saban & Ivry, 2021). We then invited these individuals to participate in our experiment. Control participants were recruited via advertisements circulated on internet forums (e.g., craigslist). Although we have limited demographic data on the control group, we assume they are selected from a similar geographic distribution as the CD group given that the only restriction was that they reside in the United States. Participants were compensated with either $20 or a $20 Amazon gift card for their participation.

Table 1 provides demographic, diagnostic, neurological, and neuropsychological information for each individual in the CD group, along with summary information for the control group. For the purposes of this study, we included individuals who had a diagnosis of cerebellar ataxia based on clinical exam (and MRI evidence as indicated by self-report) or diagnosis of spinocerebellar ataxia based on genetic testing and/or family history. We excluded individuals with Friedrich’s ataxia. For the neurological exam of ataxia, we used the Scale for the Assessment and Rating of Ataxia (SARA; Schmitz-Hübsch et al., 2006), which has a total possible score of 40 (maximum impairment). The test was modified for online administration. Specifically, items assessing gait, stance, and sitting were scored using a flow chart of questions rather than through direct observation, given concerns about remote testing. To establish general neuropsychological status, we used the Montreal Cognitive Assessment (MoCA; Nasreddine et al., 2005). For online testing, we eliminated the trail making item. Although the maximum score on the modified MoCA is 29, the scores reported below were adjusted so that they fall on a 30-point scale. Participants in the control group were also administered the MoCA.

**Table 1.** Demographic and clinical assessment for cerebellar degeneration patients (p01–p17) and healthy controls.

ID	Hand	Age	Sex	MoCA	SARA	Years of education	Etiology	Included
p01	R	48	F	29	4	25	SCA-1	Yes
p02	L	55	F	27	10	18	SAOA	Yes
p03	N/A	48	F	N/A	N/A	22	SCA-3	Yes
p04	R	51	F	26	2	12	SCA-6	Yes
p05	R	39	F	27	13	16	SAOA	Yes
p06	R	89	F	19	15	12	SCA-3	No
p07	R	80	F	26	18.5	12	SAOA	Yes
p08	R	79	M	22	8	16	SCA-6	No
p09	R	57	M	29	6	20	SCA-3	Yes
p10	R	42	F	29	10	14	SAOA	Yes
p11	R	67	M	26	15	20	SCA-5	No
p12	R	42	F	28	20	19	SAOA	Yes
p13	R	48	F	27	10	20	SAOA	Yes
p14	R	81	M	24	10	16	SAOA	Yes
p15	R	61	F	22	7	12	SCA-6	Yes
p16	L	53	F	27	17	18	SAOA	Yes
p17	R	59	F	27	9	12	SCA-6	Yes
Control (*M*)	–	52.44	–	26.39	–	16.25	–	–
Control (*SD*)	–	11.86	–	2.72	–	2.54	–	–
Patient (*M*)	–	60.5	–	25.93	10.64	16.63	–	–
Patient (*SD*)	–	15.31	–	2.83	5.36	3.86	–	–

*Note*. MoCA scores are out of a total of 29 points (perfect score). SARA scores are out of a total of 40 points (most severe ataxia). MoCA = Montreal Cognitive Assessment; SARA = Scale for the Assessment and Rating of Ataxia; SCA = spinocerebellar ataxia; SAOA = spontaneous adult-onset ataxia. Participants p06, p08 and p11 were excluded from the final sample due to their poor performance on the semantic evaluation task.

Three individuals in the CD group (p06, p08, p11) and one control (c05) were not included in the final analysis because of poor performance on the sentence verification task (overall accuracy below 70% on meaningful and meaningless sentences). Although this criterion may eliminate individuals with the most severe language problems, we were concerned it was more reflective of disengagement with the task or failure to understand the task instructions. Indeed, the mean accuracy score for these three was well below that of any of the other participants, with two performing poorly on the meaningful sentences and one responding “meaningful” to all of the meaningless sentences. For the remaining 14 individuals in the CD group, the mean score on the SARA test was 10.64 (*SD* = 5.36), a value that corresponds to mild-to-moderate impairment. At the time of testing, an average of 6.8 yr (*SD* = 7.6) had elapsed since diagnosis of cerebellar ataxia, and the participants reported symptom onset preceded diagnosis by about 4 yr.

We selected individuals for the control group by referencing our database to produce matches in terms of age and years of education (yoe). As can be seen in Table 1, the group means were comparable for the CD (*M* age = 60.5; *M* yoe = 16.63) and control (*M* age = 52.44; *M* yoe = 16.25) groups, and there was no statistically significant difference between the groups for either years of education (*t*30 = 0.34, *p* = 0.74) or age (*t*30 = 1.733, *p* = 0.09).

Although not part of our recruitment criteria, there was no significant difference between the CD (*M* = 25.93, *SD* = 2.83) and control (*M* = 26.39, *SD* = 2.72) groups on the MoCA test (*t*30 = 0.49, *p* = 0.63). Note that a MoCA score below 26 is indicative of possible cognitive impairment. Three of the CD participants in the final sample scored below this criterion as did six of the control participants. Given our focus on one important aspect of cognition, language, we did not exclude participants based on their MoCA score. Moreover, there was no relationship between the overall MoCA score and two performance metrics: task accuracy (*R* = 0.28 *p* = 0.12) and reaction time (*R* = 0.01, *p* = 0.59).

#### Procedure

Participants were recruited via an email sent to individuals in our participant database. The email provided a brief overview of the experimental goal and requirements (e.g., English as first language, completion of the 2 min (62 trial) experiment in a single session). A link was provided to the experiment and participants were informed that they could initiate testing whenever they were ready since the testing was done in an automated manner. Each participant was provided with a unique link, the code that allowed us to match the participant with their experimental data.

The link took the participant to the Gorilla platform where the participant worked through a series of introductory screens that culminated in a page where they could provide electronic informed consent. Once this was obtained, the CD and control participants completed a 2-min task designed to provide a brief assessment of American English fluency. We modified the English LexTALE task (Lemhöfer & Broersma, 2012) for our online platform. On each trial, a fixation screen was presented for 250 ms, followed by a letter string. The participant indicated if the letter string formed an English word (press “J” on keyboard) or did not form an English word (press “F”). If a response was not entered within 2,000 ms, the trial was recorded as “incorrect” and the program moved to the next trial. There were 62 trials, each with a unique target word (32 words, 32 non-words). All participants performed at a high level (overall *M* = 96%, range = 70%–100%).

Following the fluency assessment, the program moved on to the semantic verification task. The general procedure was similar to that employed in Moberget et al. (2014). The start of each trial was marked by the presentation of a fixation screen for 500 ms (Figure 1B). This was followed by the serial presentation of the sentence stem (e.g., “The man loosened the tie around his”), with each word displayed on the screen for 500 ms. We opted to use a serial presentation mode to minimize demands on eye movements. Following the presentation of the stem, the fixation screen re-appeared for 500 ms, cueing the participant that the next screen would display the target word. The target word was then presented and the participant made a speeded response, indicating if the target word resulted in a meaningful sentence (e.g., “neck”) or non-meaningful sentence (e.g., “banana”). Responses were made by pressing the “J” key with the right index finger or “F” key with the left index finger for meaningful and non-meaningful sentences, respectively. The target word remained visible until a response was detected or 2 s elapsed. The screen then went blank for 500 ms before the onset of the next trial. Participants were instructed that the primary dependent variable was response time; as such, while they should seek to achieve a high level of accuracy, they should respond as quickly as possible.

Participants first completed a practice block of 24 trials in which 16 of the sentences were meaningful and eight were non-meaningful. This was followed by five test blocks of 64 trials each, or a total of 320 test trials. Across the 320 trials, there were 80 sentences for each of the four conditions (Figure 1A), providing our assessment of prediction (high vs. low cloze probability) and CoRT (dynamic vs. static inference). Subject to this constraint, the stimulus set for each participant was based on random selection (without replacement) from the full set of 344 sentences, with the unselected items used in the practice trials. Within each of the four conditions, the target word was the final word for that sentence in the stimulus set on 70% of the trials (and, thus, formed a meaningful sentence). For the other 30% of the trials, the target word was determined by randomly generating a target word from all other target words. The experimenter then manually checked that the target word that was randomly chosen was subject to the constraint that it formed a non-meaningful sentence.

Each block took approximately 8 min and summary feedback in the form of percent correct for the previous block was provided at the end of each block, along with a reminder of the keyboard mappings. The participant moved on to the next block by pressing the spacebar. Upon completing the sentence verification task, the participant was asked to fill out a brief feedback form concerning their experience with the online testing platform. The entire experiment took approximately 50 min (longer if participants took longer breaks).

We did not include reaction times for the non-meaningful trials or for trials in which the response was incorrect. The mean values were then entered in a 2 (Group) × 2 (Prediction) × 2 (CoRT) within-subject analysis of variance (ANOVA).

## RESULTS

**Figure 2.**
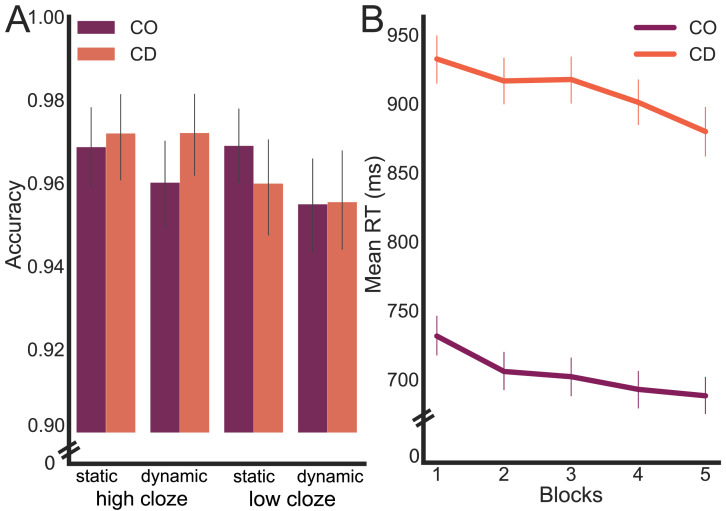
Behavioral performance summary for the cerebellar degeneration (CD) and control (CO) groups. (A) Mean accuracy averaged across blocks is close to ceiling performance for both groups. (B) Mean reaction time (RT) on correct trials for meaningful sentences as a function of test block (64 trials/block). The CD group responded slower than the CO group, with both groups showing a reduction in RT over the experimental session.

As expected, mean accuracy was very high for both groups (CD (*n* = 14): *M* = 0.97, *SD* = 0.05; control (*n* = 18): *M* = 0.96, *SD* = 0.07) across all four conditions (Figure 2A). While recognizing the limited value of an error analysis given that performance was near ceiling, we employed a three-way within-subject ANOVA with the factors (Group) × 2 (Prediction) × 2 (CoRT). None of the main effects was significant (Group: *F*_1,120_ = 0.04, *p* = 0.85, *ηp*^2^ < 0.001; Prediction: *F*_1,120_ = 0.46, *p* = 0.51, *ηp*^2^ < 0.01; CoRT: *F*_1,120_ = 0.48, *p* = 0.49, *ηp*^2^ < 0.01). The Group factor did not interact with either Prediction (*F*_1,1200_ = 0.29, *p* = 0.59, *ηp*^2^ < 0.001) or CoRT (*F*_1,120_ = 0.19, *p* = 0.66, *ηp*^2^ < 0.001), and the three-way interaction was not significant (*F*_1,120_ = 0.0001, *p* = 0.99, *ηp*^2^ < 0.001).

We analyzed the meaningful and meaningless trials separately in the analyses of the reaction time data, restricting these analyses to the trials in which the participants were accurate. For the meaningful sentences, the CD group was slower than the control group by 206 ms (main effect of group: *F*_1,30_ = 23.34, *p* < 0.001, *ηp*^2^ = 0.44). As can be seen in Figure 2B, participants became slightly faster over the course of the five test blocks (*F*_4,120_ = 5.54, *p* < 0.001, *ηp*^2^ = 0.16), but the difference between the two groups was maintained. Given the absence of a Group × Block interaction (*F*_4,120_ = 0.19, *p* = 0.94, *ηp*^2^ < 0.001), we collapsed the data across the five blocks in the subsequent analyses.

**Figure 3.**
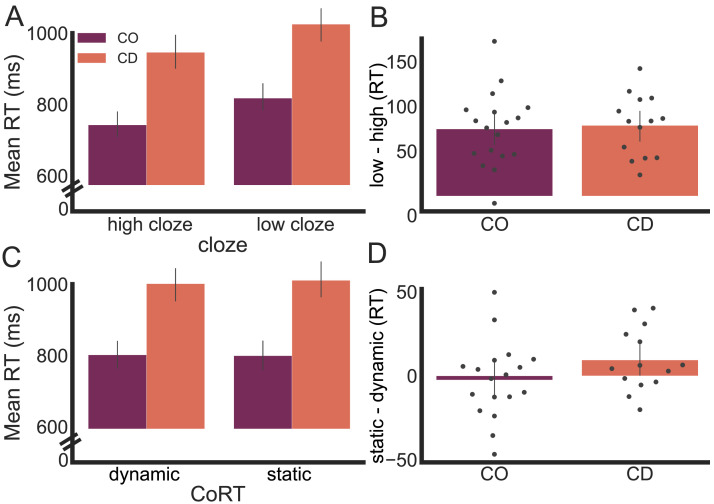
No differences were observed between the cerebellar degeneration (CD) and control (CO) groups in their ability to use predictive semantic information. (A) Mean response times (RTs) to target words as a function of cloze probability. (B) Benefit on verification task to target words that are highly predictable (low cloze–high cloze). Both the CD and CO groups were faster when the target word had high predictability, and the benefit was similar for the two groups. (C) Mean RTs to target words as a function of the dynamics conveyed by the base phrase. (D) There was no difference in RT for the dynamic and static sentences, a null effect that was similar for both groups. RTs in all graphs are limited to trials in which the participants correctly verified that the sentence was meaningful. CoRT = continuous representational transformation.

Our main interest centers on how the semantic judgments were influenced by the semantic content of the base phrases. Specifically, we asked how the degree of prediction and dynamic simulation influenced participants’ response times on the verification task. We first focused on the meaningful sentences given that the time to process the final word should be faster when that word is predicted by the context. There was a main effect of Prediction (*F*_1,120_ = 12.55, *p <* 0.001, *ηp*^2^ = 0.09), with faster response times on trials in which the target word had high cloze probability compared to trials in which the target word had low cloze probability (Figure 3A and 3B; Figure S1). Importantly, the Group × Prediction interaction was not significant (*F*_1,120_ = 0.008, *p* = 0.93, *ηp*^2^ < 0.001): Participants in the CD group showed a similar benefit to that of the control participants when the base phrase enabled the anticipation of the target word. Thus, this analysis fails to support the prediction derived from the internal model hypothesis, namely, that the difference between high and low cloze conditions would be attenuated in the CD group.

Turning to the CoRT hypothesis, we next turned to the comparison of verification times when the base phrase described a dynamic or static context. As noted in the Introduction, we had no a priori reason to suppose that verification times for the control group would be sensitive to the relative dynamics; our focus was on whether the CD group would be selectively affected on the dynamic sentences. For both groups, the results indicate that response times did not differ for sentences that described a dynamic event compared to sentences that described a static event (*F*_1,120_ = 0.02, *p* = 0.89, *ηp*^2^ < 0.001, Figure 3C and 3D; Figure S1 in the Supporting Information, available at https://doi.org/10.1162/nol_a_00083). Importantly, this factor did not interact with Group (*F*_1,120_ = 0.07, *p* = 0.79, *ηp*^2^ < 0.001). Thus, the results fail to support the hypothesis that the integrity of the cerebellum may be disproportionately important when the semantic content engages a dynamic mental transformation. The three-way Group × Prediction × CoRT interaction was also not significant (*F*_1,120_ = 0.04, *p* = 0.85, *ηp*^2^ < 0.001).

**Figure 4.**
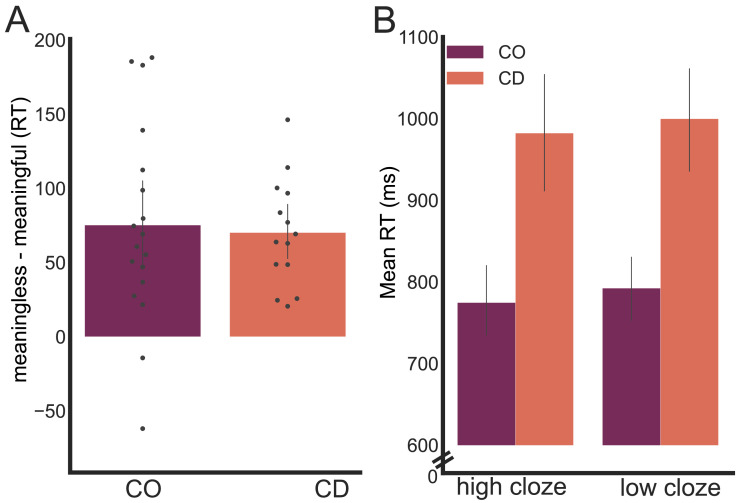
No differences observed between the cerebellar degeneration (CD) and control (CO) groups in evaluating sentences that entail prediction violations (i.e., meaningless trials). (A) Response time (RT) difference between meaningless and meaningful sentences. Participants were slower when the target word did not fit with the context, and this effect was similar for the CO and CD groups. (B) When the target word did not fit with the context, mean RT was numerically (but not statistically) faster when the context allowed for strong expectation (target word would have been high cloze). The magnitude of this effect was similar for the two groups.

We also examined the meaningless sentences to ask how the two groups responded to violations of semantic predictions. To address this question, we used a three-way ANOVA, with the factors Group, Prediction, and Trial Type (meaningful vs. meaningless). Response times were longer to target words that rendered the sentence meaningless compared to target words that resulted in a meaningful sentence (Figure 4, *F*_1,120_ = 12.85, *p* < 0.01, *ηp*^2^ = 0.097). There was no main effect of Prediction for meaningless trials (*F*_1,60_ = 0.39, *p* = 0.54, *ηp*^2^ < 0.01) and Trial Type did not interact with Group (*F*_1,120_ = 0.015, *p* = 0.91, *ηp*^2^ < 0.001) or Prediction (*F*_1,120_ = 1.95, *p* = 0.17, *ηp*^2^ < 0.001). Thus, while prediction violations slowed response times, the results failed to show that this effect was sensitive to the degree of prediction conferred by the base phrase and, importantly, failed to identify any difference in how the CD and control groups respond to violations of semantic expectancies. (We note that although the target words were selected to yield semantic predictions, a few of the violations were syntactic rather than semantic. However, there were not enough syntactic violations to do a separate analysis of these sentences.)

## DISCUSSION

To determine whether the cerebellum plays an important role in semantic processing, we tested patients with CD and age- and education-matched healthy controls on a sentence verification task. Specifically, we were interested in evaluating two hypotheses concerning how the cerebellum might contribute to semantic processing. One hypothesis was based on the idea that the cerebellum forms internal models. Extending this idea in the language domain, we hypothesized that the integrity of the cerebellum would be required to generate predictions based on semantic content. The second hypothesis is based on the idea that the cerebellum is essential for mental operations that entail a CoRT. When applying this constraint to language, we hypothesized that the integrity of the cerebellum would be essential for linguistic predictions that require a dynamic, rather than static, simulation of semantic content. The results failed to provide support for either hypothesis.

We parametrically manipulated semantic predictability (high vs. low cloze probability), and the degree to which the semantic content suggested simulation (dynamic vs. static). We observed a main effect of prediction such that participants were faster to respond to sentences in which the target word was strongly predictable from the base phrase compared to sentences in which the target word was weakly predictable. This result corroborates previous literature that has reported an influence of cloze probability on reaction time (Staub et al., 2015). Despite this evidence that the task was sensitive to the operation of predictive mechanisms, the individuals with cerebellar degeneration showed response time advantage for the high cloze words. We also did not find support for the more constrained CoRT hypothesis, as we did not observe a selective cost for the CD group in response times to the sentences that might be expected to generate dynamic simulations. Moreover, the results provide indirect evidence against another task-independent hypothesis of cerebellar function, error-based processing (Fiez et al., 1992). Participants were slower to respond to the meaningless sentences than to meaningful sentences (D’Mello et al., 2017). Taking this as a measure of sensitivity to prediction violations, the CD and control groups showed a similar increase in response time in judging a sentence as meaningless.

Using similar tasks, neuroimaging (D’Mello et al., 2017; Lesage et al., 2017; Moberget et al., 2014) and neurostimulation studies (D’Mello et al., 2017; Lesage et al., 2012) have produced evidence to suggest a cerebellar role in semantic prediction, with the emphasis in those studies on the internal model hypothesis. For example, compared to scrambled word strings, predictive sentences produce an increase in the BOLD response in a circumscribed region of posterolateral right cerebellum, and violations of these predictions produce a strong response that is roughly symmetric for right and left posterolateral cerebellum (Moberget et al., 2014). These results stand in contrast to the null results of the present study, one in which we set out to provide a more direct test of a general and constrained variant of the internal model hypothesis through our parametric manipulations.

Taken together, we find an unsatisfying misalignment of neuroimaging, neurostimulation, and neuropsychology findings in terms of how the cerebellum contributes to semantic processing. It is no doubt foolhardy to wish for a perfect marriage; different methods have their strengths and weaknesses. With respect to the cerebellum and cognition, much of the impetus has come from the neuroimaging literature, a method that is quite powerful in identifying areas that show activity correlated with tasks and/or processes. Neuropsychology offers a powerful tool to test functional hypotheses, although neural plasticity places some limitations in interpretation, especially for null results. It is also important to keep in mind one important limitation with cerebellar neuroimaging: The BOLD signal appears to be a function of activity at the mossy fiber terminals (Mathiesen et al., 2000) rather than activity reflective of intra-cerebellar processing (e.g., Purkinje cell firing). Thus, cerebellar neuroimaging is providing a window concerning the inputs to the cerebellum, but mute in terms of how that information is transformed by the cerebellum.

Caution is, of course, warranted when interpreting null results. We can consider a few possible explanations for why we failed to observe support for either hypothesis. First, we do note that there were performance differences between the CD and control groups, namely, that the CD group was slower overall. This is to be expected given their ataxia. In previous studies involving manual responses for choice RT tasks, we have observed an increase in reaction time of between 50 and 150 ms (Breska & Ivry, 2018; Helmuth et al., 1997). In the present study, the increase was 220 ms. It is possible that the larger difference in the present study is motoric in nature, related to the fact the testing was performed online with a non-optimal response device (computer keyboard) for individuals with ataxia. On the other hand, it is possible that the increase in response time does reflect an impairment in sentence comprehension or some more generic aspect of cognitive processing, and that our experimental manipulations are insensitive to this impairment.

Second, our patient sample is quite heterogeneous, composed of individuals with different etiologies. It is possible that, when treated as a group, the pathology does not consistently impact critical regions within the cerebellum that are essential for semantic predictions (e.g., right posterolateral cerebellum). However, it is unlikely that the null results are attributable to divergent patterns of degeneration as the literature indicates that pathology in posterolateral regions of the cerebellum are generally associated with the etiologies of our sample (Hernandez-Castillo et al., 2018; Lukas et al., 2006). Nonetheless, it would be useful to recruit a larger sample size in future work to allow for voxelwise morphometry to relate patterns of pathology and behavior (Hernandez-Castillo et al., 2018; Kansal et al., 2017). We were not able to do any analysis along these lines in the present study: Not only is our sample small, but also we do not have scans for most of the participants. Another approach would be to recruit individuals with unilateral lesions encompassing posterolateral regions and compare the effect of right and left cerebellar lesions.

Another approach might be to focus on individuals with cerebellar degeneration who show language impairments on standard neuropsychological tests of language (e.g., range of semantic fluency tasks, word completion tasks, Boston Naming Test (Kaplan et al., 1983)). This information would allow us to ask if performance on our experimental task of semantic prediction relates to profiles of language competence as assessed by these instruments. While our screening test for cognitive impairment (MoCA) included a semantic fluency task, it is likely neither comprehensive nor sensitive enough to detect subtle language deficits. We note that the patient group in the current study presented with relatively mild cognitive symptoms (as reported by MoCA). It is possible that individuals with milder impairment are more likely to participate in online studies.

Third, our experimental design may not have been optimal for testing the prediction and/or CoRT hypotheses. For example, we imposed a 500 ms delay prior to the presentation of the target word, with this interval serving as a cue that the next word would be the target. However, the delay period may have masked the cost of an impaired prediction process, one that operates more slowly in the CD group compared to the control group. That is, the delay period could have provided a “slack” window that provided sufficient time for the CD group to generate a robust semantic prediction (Pashler, 1994). This hypothesis can be addressed by repeating the experiment without the delay period, perhaps using a change in color or font to designate the target word. In terms of the CoRT hypothesis, sentence processing in general may entail the dynamic manipulation of the constituent words and phrases, independent of the semantic content of the sentences. By this view, a CoRT impairment might impact both our dynamic and static sentences. Tasks involving word pair judgments would present another means to compare semantic comprehension without imposing the more temporally extended demands of sentence processing. For example, would the CD group show weaker priming for dynamic word pairs (ball–throw) compared to static word pairs (sunset–red).

Fourth, drawing on the motor control literature, we have conceptualized prediction as a dynamic process, one in which an input is fed into an internal model to generate an expectancy of the expected sensory consequences (i.e., the target word). It is possible that semantic expectancies do not entail simulation of this form, but rather emerge from memory retrieval (Firth, 1957; Mikolov et al., 2013; Smith & Levy, 2013). That is, after hearing the phrase, “The boss refused to give him a —,” anticipating the word “raise” may not require running a simulation; rather, “raise” may be primed because it tends to co-occur (sadly) with the words “boss” and “refused.” Indeed, a memory-based account might be especially appropriate for high cloze sentences given that they tend to be more formulaic than low cloze sentences (Conklin & Schmitt, 2012). In general, cerebellar degeneration does not impact memory retrieval (Appollonio et al., 1993; McDougle et al., 2022). The similar sensitivity of the CD and control groups to cloze probability in the current study may be another manifestation of spared memory and memory retrieval in cerebellar degeneration.

## CONCLUSIONS

Dating back to the earliest neuroimaging studies, language studies have figured prominently in the quest to understand the role of the cerebellum in cognition. Many of the studies have involved tasks that tax semantic retrieval, with the results showing consistent activation in right posterolateral cerebellum when people generate semantic associates (Petersen et al., 1989), manipulate information in verbal working memory (Marvel & Desmond, 2010), or verify meaningfulness (D’Mello et al., 2017; Moberget et al., 2014). Given that cerebellar pathology is not associated with profound aphasia, functional hypotheses have focused on how the cerebellum facilitates linguistic processing. Prediction has been central to most of these hypotheses given that fluent communication requires feedforward processing, defined within the domain of language comprehension as the ability to anticipate linguistic intent that arises during a conversation with another individual or reading a text. The internal model and CoRT hypotheses provide two ways in which the cerebellum might support prediction in the domain of language, and indeed, support cognition more generally (Diedrichsen et al., 2019; McDougle et al., 2022). However, we failed to obtain support for either hypothesis when put to what we see as rigorous tests. We have outlined limitations with our study, and certainly see a need for future studies that provide further tests of the internal model and CoRT hypotheses, as well as other dimensions of semantic processing. We also recognize that prediction, at least as conceptualized here, may not be the appropriate kernel for understanding how the cerebellum supports language.

## ACKNOWLEDGMENTS

Thanks to Jonathan Tsay and Amanda LeBel for providing helpful feedback on the manuscript.

## FUNDING INFORMATION

Richard B. Ivry, National Institutes of Health (https://dx.doi.org/10.13039/100000002), Award ID: NS092079. Richard B. Ivry, National Institutes of Health (https://dx.doi.org/10.13039/100000002), Award ID: NS105839.

## AUTHOR CONTRIBUTIONS

**Maedbh King**: Conceptualization: Equal; Data curation: Equal; Formal analysis: Equal; Investigation: Equal; Methodology: Equal; Project administration: Equal; Software: Lead; Supervision: Equal; Validation: Equal; Visualization: Equal; Writing—original draft: Equal; Writing—review & editing: Equal. **Sienna Bruinsma**: Data curation: Equal; Formal analysis: Equal; Methodology: Equal; Visualization: Equal; Writing—original draft: Equal; Writing—review & editing: Supporting. **Richard B. Ivry**: Conceptualization: Equal; Funding acquisition: Lead; Project administration: Equal; Supervision: Equal; Writing—original draft: Equal; Writing—review & editing: Equal.

## COMPETING INTERESTS

Richard B. Ivry is a co-founder with equity in Magnetic Tides, Inc. The other authors declare no competing interests exist.

## DATA AVAILABILITY STATEMENT

All of the sentences used across experimental task blocks (including cloze and CoRT ratings) are available to download on Open Science Framework at https://osf.io/aegkm/?view_only=None. The code used to analyze these data is available to download on Github at https://github.com/maedbhk/NOL_cerebellum.

## Supplementary Material



## TECHNICAL TERMS

Internal model: Model that represents the dynamic properties of an object (e.g., body part, tool), allowing the agent to anticipate the sensory consequences of an action within a particular environment.
Cloze probability: Linguistic metric that quantifies the context-dependent predictability of a word.
Continuous operations of representational transformation (CoRT): A theory proposing that the cerebellum facilitates mental processing when an internal representation undergoes a continuous and dynamic transformation but is not essential when the mental operations entail more discrete or transformations maintenance of static representations.
Spinocerebellar ataxia (SCA): Progressive and degenerative hereditary disorder that results in atrophy of the cerebellum. Symptoms typically include uncoordinated gait, loss of balance, and cognitive deficits.
