# Supplementary material for: No Evidence for Semantic Prediction Deficits in Individuals With Cerebellar Degeneration
Source: Neurobiol Lang (Camb). 2024 Aug 15;5(3):635–51. doi: 10.1162/nol_a_00083 (PMC11338309; doi:10.1162/nol_a_00083)
Supplement: Supplementary file 1 [file nol-5-3-635-s001.pdf]

Supplemental Materials

Sentence	CoRT Rating	Cloze	Trial Type
1. The farmer collected wood to build a new barn	Dynamic	Low	Meaningful
2. They were startled by the sudden noise	Dynamic	High	Meaningful
3. Drake didn't like meat because of the taste	Static	Low	Meaningful
4. His boss refused to give him a raise	Static	High	Meaningful
5. The driver drove too quickly around the corner	Dynamic	Low	Meaningful
6. She roasted the marshmallow over the fire	Dynamic	High	Meaningful
7. His worst fear was that the spectators would boo	Static	Low	Meaningful
8. The patient's broken arm took a while to heal	Static	High	Meaningful
9. The teacher wrote the problem on the game	Dynamic	High	Meaningless
10. She wanted a great job so she could get bear	Static	Low	Meaningless

Table S1. Example sentences indicating CoRT and Cloze ratings. Sentences 1-8 are examples of meaningful sentences, while sentences 9 and 10 are meaningless. All sentences used in the experiment (including CoRT and cloze ratings) can be downloaded at https://osf.io/hb3w8?view_only=ad29ec1a58e94e9a92ee120e031a0016.

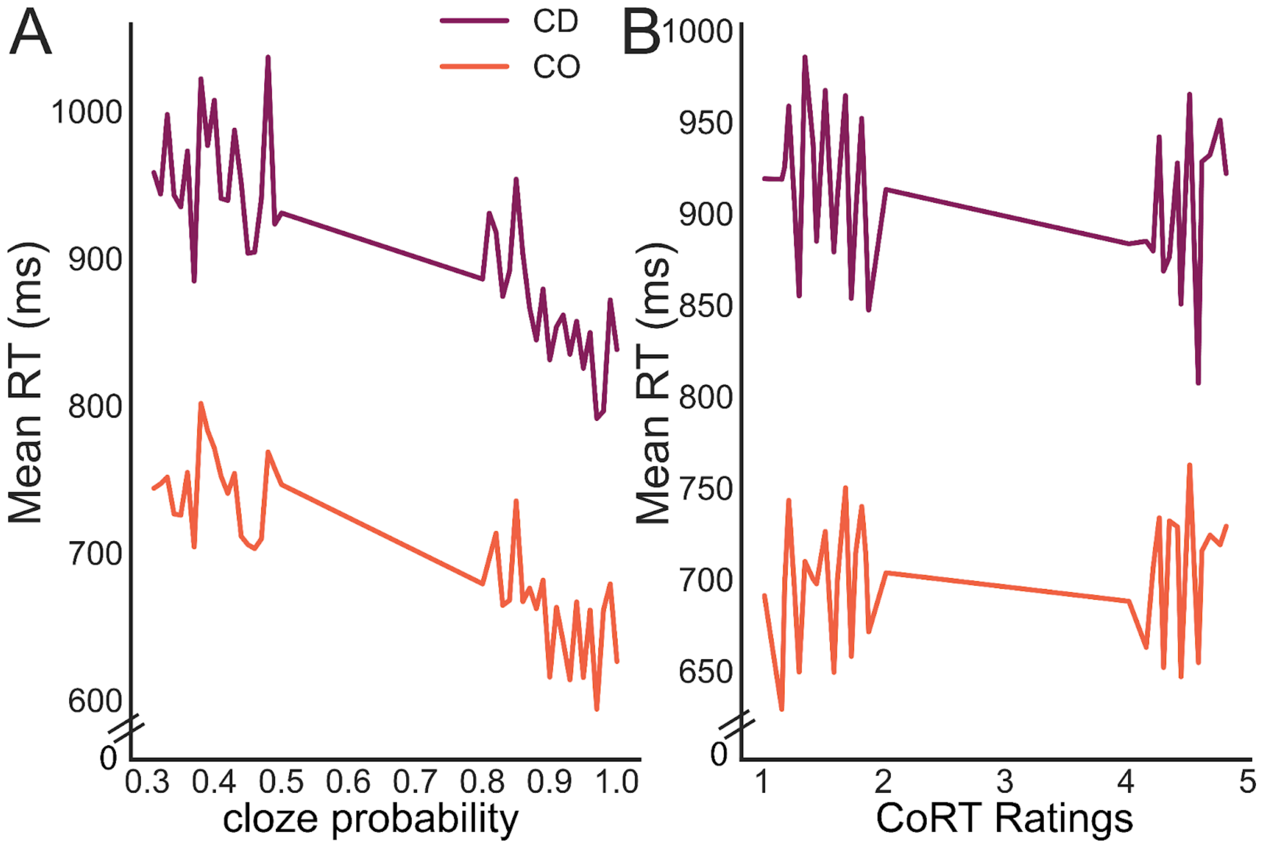

Fig S1. ***Reaction time performance across cloze (A) and CoRT (B) ratings for the CD and CO groups.*** *Same data as shown in Figure 2, but binned as a function of (A) cloze probability or (B) CoRT rating (where high values correspond to sentences rated as most dynamic. The test stimuli were limited to sentences with a mean cloze probability of 0.3 - 0.5 (low cloze) or 0.8 - 1.0 (high cloze). Inclusion in the test set was limited to sentences with a CoRT ratings 1.0 – 2.0 (static, non-CoRT) or 4.0 – 5.0 (dynamic, CoRT)*
